# Effect of Flow Rate of Side-Type Orifice Intake on Withdrawn Water Temperature

**DOI:** 10.1155/2014/979140

**Published:** 2014-06-11

**Authors:** Xueping Gao, Guangning Li, Yunpeng Han

**Affiliations:** State Key Laboratory of Hydraulic Engineering Simulation and Safety, Tianjin University, Tianjin 300072, China

## Abstract

Side-type orifice intake is a type of selective withdrawal facility used in managing reservoirs to mitigate the negative effects of low-temperature water. Based on the temperature data of a thermal stratified reservoir in China, an experiment was conducted in flume to study the influence of intake flow rate on withdrawn water temperature with different temperature distributions. Results indicated that withdrawn water temperature changed with different flow rates. The temperature change was determined by the water temperature gradients above and below the intake, whereas the change trend of temperature depended on the difference between the water temperature gradient above and below the intake. We likewise proposed a new equation with which the withdrawn water temperature of a thermal stratified reservoir using a side-type orifice could be calculated. These findings could be directly applied to the design and operation of side-type orifice intake in thermal stratified reservoirs.

## 1. Introduction

Most reservoirs are typically temperature stratified as a result of surface heating and wind mixing. Low-temperature water withdrawn in a traditional manner from deep reservoirs is unsuitable for irrigation and may easily cause a hazard to aquatic organisms living downstream. Thus, water should be released from different depths (i.e., temperature), which is the core concept of selective withdrawal [1].

Stratified flow occurs when water is withdrawn due to the changes in fluid density caused by differences in temperature [2]. Over the past 60 years, numerous studies had applied numerical simulations or physical experiments to investigate withdrawal with stratified flow. For the physical experiments, a number of studies were conducted on flow through the orifices or bottom slots within a two-layer system [3–7]. Several hydraulic analyses and extended theories of selective withdrawal for both 2D and 3D flows in a two-layer flow were proposed [8, 9]. A system with linear stratification was likewise often adopted. Experiments on linearly stratified flow through orifices in vertical walls were performed to study the withdrawal layer thickness [10–12].

However, none of the previous experiments were conducted with a nonlinear continuous temperature distribution similar to the practical temperature condition in reservoirs. Little information is available on the effect of intake flow rate on withdrawn water temperature. Based on the temperature data of a thermal stratified reservoir in southern China, we analyzed the effect of flow rate on the withdrawal water temperature of a side-type orifice intake and proposed an equation for withdrawn water temperature prediction.

## 2. Method and Material

### 2.1. Theory and Facility

For the model test of water temperature in a thermally stratified reservoir using one medium (fresh water), the temperature distribution was similar when the Froude number *Fr* and the density Froude number *Fd* are equal to those of the prototype [13]. The temperature distribution should satisfy the equation

(1) $$\begin{array}{c} \lambda_{\Delta T}=1, \end{array}$$

and the temperature difference should be the same as that in the prototype. When the base temperature (hypolimnion) of the model experiment differed from that of the prototype, the temperature of the withdrawn water was

(2) $$\begin{array}{c} {\overset{-}{T}}_{H}={\overset{-}{T}}_{M}+\left(T_{BH}-T_{BM}\right), \end{array}$$

where ${\overset{-}{T}}_{H}$ is the withdrawn water temperature of prototype, ${\overset{-}{T}}_{M}$ is the withdrawn water temperature of the model, *T*_*BH*_ is the basic temperature of prototype, and *T*_*BM*_ is the basic temperature of model.

**Figure 1:**
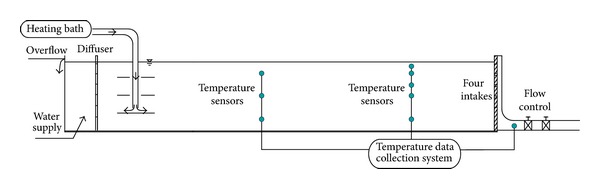
Schematics of selective withdrawal using a side-type orifice intake.

The experiment was designed based on the idea of generating a continuous temperature distribution with the same flow direction in the entire flume. A rectangular flume that was 20 m long, 2 m wide, and 0.8 m high with a horizontal bottom (Figure 1) was selected. The flume was fixed on a water feeding tank. The outflow was measured at the end of the flume. The water level in the reservoir was controlled by a weir at the far end of the flume, and the overflow water returned to the feeding tank. Aside from the flume, the experiment system contained a heating control unit, a stratifying unit, a pipeline system, and a data collecting unit. The experimental data were collected and recorded synchronously using a data collection instrument. Four intakes were placed on the vertical direction, 6 cm in height and 8 cm in width, with submersing depths of 15 cm, 23 cm, 31 m, and 39 cm, respectively, called the first to the fourth intakes with three flow rates ranging from 1.0 to 2.5 (1.0, 1.7, and 2.5 L/s).

In the preparation stage, the entire flume was divided into three layers: the bottom layer and two heating baths which were filled with water pumped from the water feeding tank. According to the original temperature of the bottom water and the objective temperature stratification, parameters were set to the heating bath, and water was automatically heated up to a certain temperature. The hot water was then poured into the flume through a special device. The device contained a large plane board, such that the water that poured onto the board would not disturb the previous layer. Meanwhile, the volume of the water pumped into the flume was strictly controlled, such that the water level in the flume rose by only 1 cm in 5 min. When the middle layer reached the appropriate elevation, pouring was stopped for a while to enable the recently poured water to be evenly mixed. Water of the top layer was poured into the flume using the same method. Finally, we could obtain a satisfactory temperature distribution.

**Figure 2:**
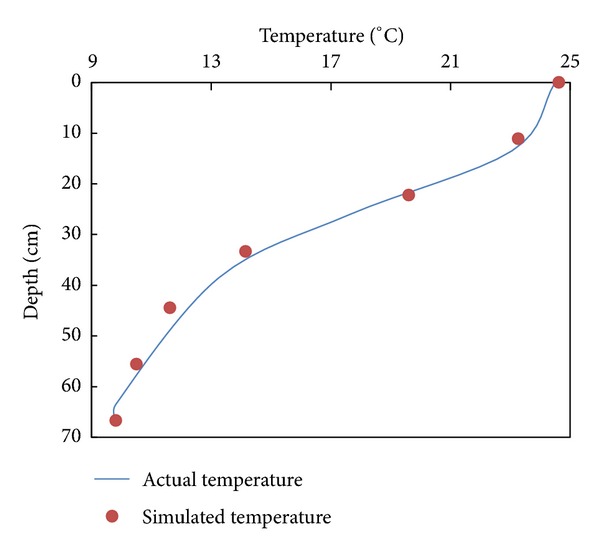
Temperature distribution in the reservoir.

**Figure 3:**
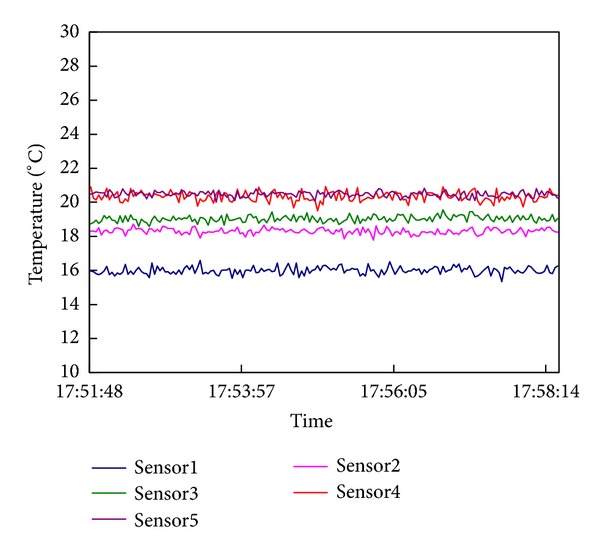
Duration curve of temperature sensors.

**Figure 4:**
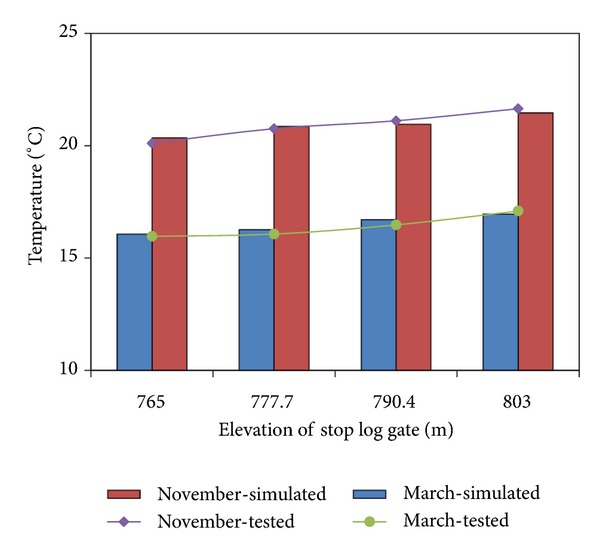
Comparison between the simulated and test values.

The simulated temperature distribution suitably matched the actual temperature distribution (Figure 2). The collected data showed that the temperature stratification near the intake would not change in the first 10 min (Figure 3). Thus, the data on withdrawn water temperature collected synchronously within 10 min were reliable and accurate. A comparison of withdrawn water temperature between the model test and numerical simulation (using EFDC) is shown in Figure 4. The comparison proved the feasibility and reliability of our experiments.

### 2.2. Experiment Conditions

**Figure 5:**
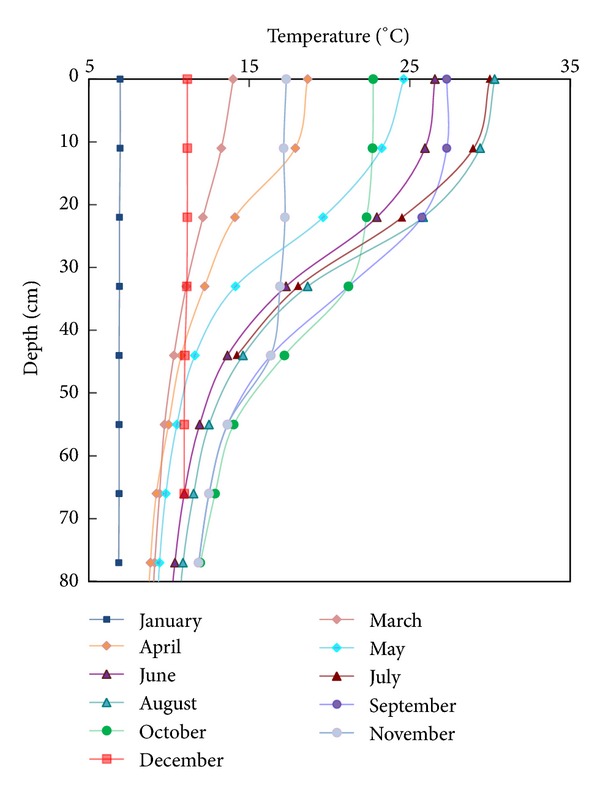
Schematics of temperature distribution.

Experiments were conducted based on the temperature data of a thermal stratified irrigation reservoir in Jiangxi Province, China.  Figure 5 shows the observed temperature stratification in the reservoir during a typical reference year. From early spring in March to late autumn in October, the temperature differences between the top and bottom of the reservoir were significant because of the high temperature and strong sunshine. In winter, the temperature rapidly dropped, thus decreasing the water temperature in the top region, in which the temperature is typically higher than that in the bottom throughout the year. For the initial temperature profile in the flume, the length scale in the vertical direction was 1 : 18.

**Figure 6:**
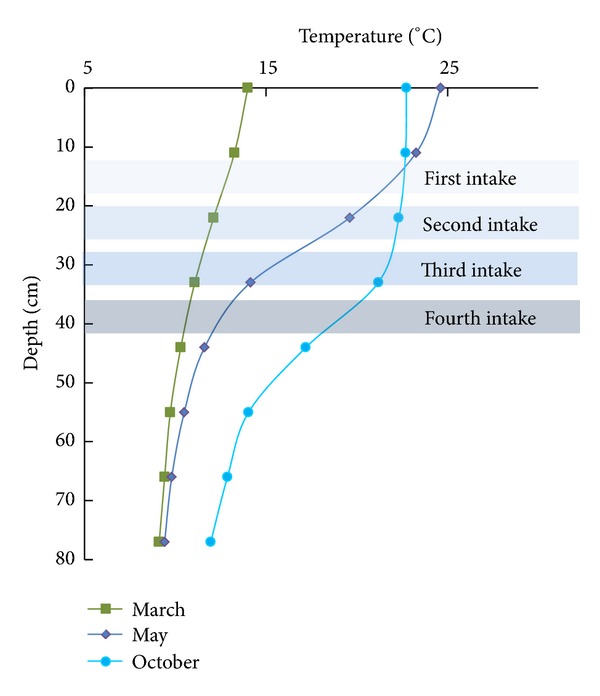
Location of intakes in the experiments.

We selected three typical stratifications, as shown in Figure 6. Each distribution had its own features within the layer intakes. In March, the temperature difference between the top and bottom was approximately 5°C. The temperature exhibited a general linear increment up to the increase in elevation. For the distribution in October, the temperature gradient decreased when the elevation increased. In May, the temperature gradient initially increased, but when a higher elevation was reached, the gradient began to decrease. The temperature differences in May and October were 15°C and 10°C, respectively.

## 3. Results and Discussion

Multigroup experiments were conducted and the results under the condition of different intakes and flow rates are shown in Table 1.

With a certain temperature stratification and submersing depth, the temperature of withdrawn water changed with the flow rate. For the temperature distribution in March, the temperature of the withdrawn water from each intake increased when the flow rate increased, but the increase amplitude was within a very small range from 0.12°C to 0.24°C. In May, each intake had its own change law. For the first and the second intakes, the withdrawn water became colder when the flow rate increased. The temperature of withdrawn water decreased by 0.24°C and 0.31°C, respectively, when the flow rate changed from 1.0 L/s to 2.5 L/s. For the third and the fourth intakes, the withdrawn water became hotter when the flow rate increased. The temperature of the third intake increased from 15.58°C to 16.17°C when the flow rate changed from 1.0 L/s to 2.5 L/s, whereas the temperature of the fourth intake increased from 12.77°C to 12.96°C at the same situation. In terms of the four experiments in October, the temperature of withdrawn water decreased when the flow rate increased. The change amplitude was between 0.15°C and 0.35°C.

**Figure 7:**
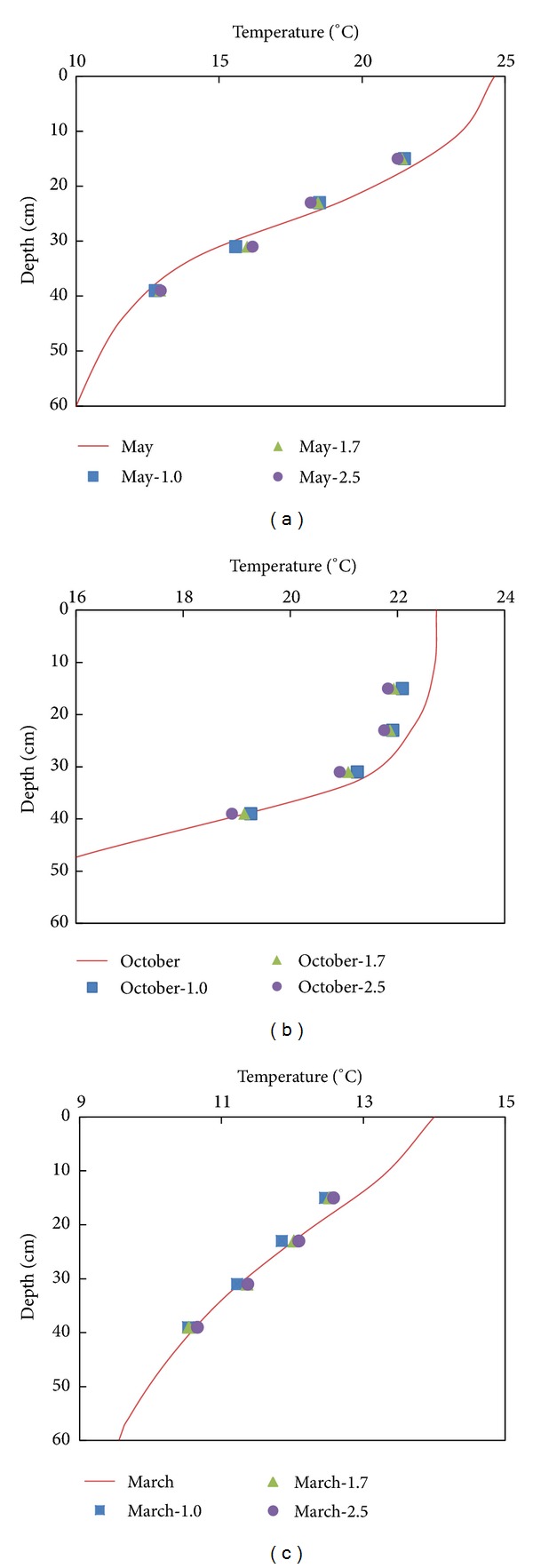
Comparison between the withdrawn water temperature and temperature of water at the orifice center in each month ((a)–(c)).

Figure 7 shows that the relationship between the withdrawn water temperature and the water temperature at the orifice center is likewise determined by the temperature distribution near the orifice. When the temperature distribution underwent a uniform change in the vertical direction, similar to the case of the second to the fourth intakes, the withdrawn water temperature showed good agreement with the water temperature of the orifice center. If the temperature distribution was similar to that in October and the top half in May, the withdrawn water temperature was certainly less than the water temperature at the orifice center. When the temperature gradient near the intake gradually decreased, the withdrawn water temperature would be higher than the water temperature at the orifice center.

According to this phenomenon, we propose an equation to predict the withdrawn water temperature as follows:

(3) $$\begin{array}{c} D_{a}=D_{b}=0.53Q^{1/3}{\left(\frac{g\left(\rho_{b}-\rho_{s}\right)}{\rho_{b}\cdot H}\right)}^{-1/6}\\ T_{w}=\left(\frac{T_{a}+T_{b}}{2}-T_{0}\right){\left(\frac{H-H_{0}}{H}\right)}^{1/2}+T_{0}, \end{array}$$

where *D*_*a*_ is the thickness of water layer above the orifice, m; *D*_*b*_ is the thickness of water layer below the orifice, m; *Q* is the flow rate, m^3^/s; *ρ*_*s*_ is the density of the water on the surface of the reservoir; *ρ*_*b*_ is the density of the water at the bottom of the reservoir; *H* is the total depth of the reservoir; *T*_*w*_ is the withdrawn water temperature; *H*_0_ is the depth of the orifice (from the center); *T*_*a*_ is the temperature of water with the depth of *H*_0_ − *D*_*a*_; *T*_*b*_ is the temperature of water with the depth of *H*_0_ + *D*_*b*_; and *T*_0_ is the temperature of water at the center of the orifice.

**Figure 8:**
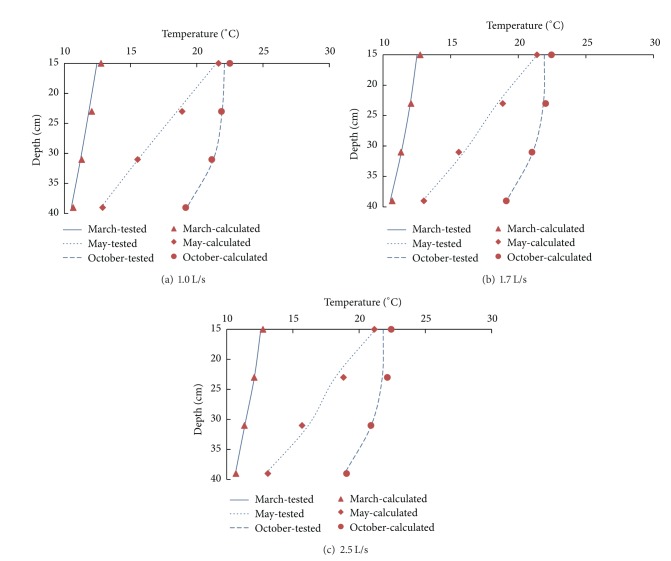
Comparison between the test value and the calculated value.

*D*_*a*_ and *D*_*b*_ were initially determined, and other factors of temperature could be obtained from the temperature distribution. The comparison showed that the difference between the test value and the calculated value was no more than 3%, as shown in Figure 8. However, the scope of the equation was limited to the reservoir with a total temperature higher than 4°C using the side-type orifice intake, and the intake should not be on the surface or at the bottom of the reservoir.

Based on the analyzed data, we observed that the temperature of withdrawn water changed with different flow rates, but the change trend depended on the temperature distribution near the intake. When the temperature distribution underwent a uniform change in the vertical direction, the influence of flow rate on withdrawn water temperature was negligible. If the gradient of the temperature in the vertical direction gradually increased, the withdrawn water temperature decreased as flow rate increased, similar to the case of the first intake in May. If the gradient gradually decreased, the withdrawn water temperature increased as the flow rate increased, similar to the situation of the third and fourth intakes in May. Combined with temperature distribution and the temperature of withdrawn water, we could easily determine that the variation of withdrawn water temperature depended on the gradient level of water temperature near the intake. For instance, with the temperature distribution of May, when the flow rate increased from 1.0 L/s to 2.5 L/s, the withdrawn water temperature of the third intake with a larger gradient increased by 0.59°C, whereas the temperature using the fourth intake increased by 0.19°C.

Aside from a discussion of the effect of flow rate on withdrawn water temperature, the engineering application was likewise considered. A study by Martin and McCutcheon [14] suggested that a difference in water temperature flow rate of 1°C shifted ice formation downstream by 100 km. With regard to the aquatic organism living downstream, some of them had an obvious critical temperature, similar to the case of fish spawning and crop tillering [15]. Thus, any increment in temperature would have a significant influence. Meanwhile, the generation benefit, which is directly related to the withdrawal elevation, should be considered as well. Thus, an intake with lower elevation should be used, if possible. The results of our study and the predictive equation could be directly applied to reservoir selective withdrawal using a side-type orifice intake for a possibility of increasing withdrawn water temperature without changing intake. Such knowledge is essential in the operation of a withdrawal intake. When the withdrawn water temperature does not meet the requirement and the difference is small, the proposed approach may be a suitable method for altering the temperature by changing flow rate, but not the intake, according to the characteristics of the temperature distribution.

## 4. Conclusion

Previous experiment studies on selective withdrawal were conducted with a single, linear density distribution. Furthermore, limited information was available on the effect of intake flow rate on withdrawn water temperature. In this paper, we successfully simulated a continuous temperature distribution in flume and conducted an experiment study. This method was proven to be effective and feasible. We studied the effect of flow rate on withdrawn water temperature and proposed a predictive equation of withdrawn water temperature.

For the side-type orifice intake, the withdrawn water temperature changed with different flow rates, and the variation rested with the gradient level of water temperature. However, the change trend of temperature depended on the gradient of water temperature near the intake. With an increment of gradient in the vertical direction, the temperature of withdrawn water decreased as the flow rate increased. Meanwhile, with a decrement of gradient in the vertical direction, the temperature of withdrawn water increased as the flow rate increased.

The selection of intake and the flow rate of withdrawal was the parameters that could be artificially controlled. With the proposed equation, an administrator could easily identify an economical and appropriate operation mode that considers both withdrawn water temperature and generation benefit.

## Figures and Tables

**Table 1:** Withdrawn water temperature with different submerse depths and discharges.

Depth (cm)	March			May			October		
	1.0 L/s	1.7 L/s	2.5 L/s	1.0 L/s	1.7 L/s	2.5 L/s	1.0 L/s	1.7 L/s	2.5 L/s
First intake (15)	12.46	12.52	12.58	21.48	21.4	21.24	22.09	21.93	21.82
Second intake (23)	11.85	12.02	12.09	18.51	18.46	18.2	21.91	21.86	21.75
Third intake (31)	11.22	11.36	11.37	15.58	15.98	16.17	21.25	21.08	20.92
Fourth intake (39)	10.53	10.54	10.66	12.77	12.94	12.96	19.26	19.14	18.91
